# Post-hypothermic cardiac left ventricular systolic dysfunction after rewarming in an intact pig model

**DOI:** 10.1186/cc9334

**Published:** 2010-11-23

**Authors:** Ole Magnus Filseth, Ole-Jakob How, Timofei Kondratiev, Tor Magne Gamst, Torkjel Tveita

**Affiliations:** 1 Department of Anesthesiology, Institute of Clinical Medicine, University of Tromsø, N-9037 Tromsø, Norway; 2 Department of Medical Physiology, Institute of Medical Biology, University of Tromsø, N-9037 Tromsø, Norway; 3 Department of Anesthesiology, University Hospital of Northern Norway, N-9038 Tromsø, Norway; 4 Department of Cardiothoracic surgery, Institute of Clinical Medicine, University of Tromsø, N-9037 Tromsø, Norway

## Abstract

**Introduction:**

We developed a minimally invasive, closed chest pig model with the main aim to describe hemodynamic function during surface cooling, steady state severe hypothermia (one hour at 25°C) and surface rewarming.

**Methods:**

Twelve anesthetized juvenile pigs were acutely catheterized for measurement of left ventricular (LV) pressure-volume loops (conductance catheter), cardiac output (Swan-Ganz), and for vena cava inferior occlusion. Eight animals were surface cooled to 25°C, while four animals were kept as normothermic time-matched controls.

**Results:**

During progressive cooling and steady state severe hypothermia (25°C) cardiac output (CO), stroke volume (SV), mean arterial pressure (MAP), maximal deceleration of pressure in the cardiac cycle (dP/dt_min_), indexes of LV contractility (preload recruitable stroke work, PRSW, and maximal acceleration of pressure in the cardiac cycle, dP/dt_max_) and LV end diastolic and systolic volumes (EDV and ESV) were significantly reduced. Systemic vascular resistance (SVR), isovolumetric relaxation time (Tau), and oxygen content in arterial and mixed venous blood increased significantly. LV end diastolic pressure (EDP) remained constant. After rewarming all the above mentioned hemodynamic variables that were depressed during 25°C remained reduced, except for CO that returned to pre-hypothermic values due to an increase in heart rate. Likewise, SVR and EDP were significantly reduced after rewarming, while Tau, EDV, ESV and blood oxygen content normalized. Serum levels of cardiac troponin T (TnT) and tumor necrosis factor-alpha (TNF-α) were significantly increased.

**Conclusions:**

Progressive cooling to 25°C followed by rewarming resulted in a reduced systolic, but not diastolic left ventricular function. The post-hypothermic increase in heart rate and the reduced systemic vascular resistance are interpreted as adaptive measures by the organism to compensate for a hypothermia-induced mild left ventricular cardiac failure. A post-hypothermic increase in TnT indicates that hypothermia/rewarming may cause degradation of cardiac tissue. There were no signs of inadequate global oxygenation throughout the experiments.

## Introduction

Physicians must take care of patients exposed to various types and levels of hypothermia. In severe accidental hypothermia, surviving victims may have an excellent prognosis, even in the most serious cases of hypothermic circulatory arrest [1,2]. Still, even if spontaneous circulation is maintained at moderate (30 to 34°C) or severe (below 30°C) body temperature [3] during accidental hypothermia, victims may present with impaired cardiovascular function. While the occurrence of life threatening cardiac arrhythmias usually subside with increasing core temperature [4], hypotension and low cardiac output may prevail during and after rewarming [5,6].

On the other hand, in the context of induced moderate hypothermia being applied as a protective measure during cardiac surgery, or as a therapeutic action to mitigate global brain ischemic injury in survivors after cardiac arrest, cardiovascular side effects from hypothermia seldom cause problems [7].

In awake homoeothermic animals exposure to cold that may lower body temperature is stressful and will lead to strong neuroendocrine activation and an increase in heart rate and blood pressure [8]. There is experimental evidence that this stress reaction may eliminate the positive effects achieved by applying therapeutic hypothermia [9,10]. As a recognition that it is the combined effect of sedation/anesthesia and hypothermia that favors both the central nervous and the cardiovascular system after cardiac arrest, deep sedation is now an integral part of therapeutic hypothermia protocols [7]. This is in accordance with clinical observations that surviving victims of accidental hypothermia influenced by sedative drugs or ethanol tolerate hypothermia and rewarming better than victims unaffected by these substances [6,11].

From experimental hypothermia research results have been somewhat confounding regarding the effect of low body temperature *per se *on myocardial function. From studies on isolated dog and rabbit hearts subjected to moderate and severe hypothermia, increased left ventricular (LV) contractility and increased cardiac work have been reported [12,13]. Core cooling to 33°C in a pig model mimicking therapeutic hypothermia suggested improved systolic, but depressed diastolic function [14]; similar results were found in surface cooled dogs [15]. In severe hypothermia, increased LV contractile force was demonstrated in intact dogs during surface cooling to 20 to 25°C [16]. Likewise, immature swine cooled by extracorporeal circuit peaked in LV stroke volume and work at 29°C [17]. On the other hand, intact dogs that were core cooled to 25°C and rewarmed showed reduced myocardial contractility during as well as after hypothermia [18]. The issue of differences in physiologic effects between species was demonstrated in a recent comparative study using cardiac tissue from humans and rabbits that revealed reduced inotropy by moderate hypothermia in human as opposed to rabbit [19]. The findings were related to differences in myocardial tissue sarcoplasmic reticulum Ca^2+ ^storage and Ca^2+ ^sensitivity [19].

The cooling mechanism in out-of-hospital accidental hypothermia is by surface cooling, and this approach has also been used to induce therapeutic hypothermia in survivors after cardiac arrest even though core cooling by indwelling vascular catheters has been developed and used over the last years [7]. Rewarming in therapeutic hypothermia is passive or by active external warming. As for victims of accidental hypothermia with a perfusing rhythm, the American Heart Association recommends the use of active external rewarming in moderate hypothermia and core rewarming in severe hypothermia [3]. However, surface rewarming by forced air has been proven to be safe and successful even in victims of severe hypothermia considered having a perfusing rhythm [20].

To our knowledge, no clinical studies have so far described the complete time-course of surface cooling, steady-state hypothermia and surface rewarming on cardiovascular function and other clinically related physiologic variables in either accidental or therapeutic hypothermia.

Previous experimental hypothermia studies of surface cooling/rewarming in animals with maintained circulation have been performed using rodents [21] and dogs [22,23]. However, in these species cardiovascular function seem to react differently to changes in temperature as rodents increase their stroke volume (SV) during severe hypothermia [24,25], whereas in dogs SV remains unchanged at this temperature zone and an elevated systemic vascular resistance (SVR) is maintained after rewarming even from prolonged surface hypothermia [22,23].

The apparently closer morphologic and physiologic relationship between humans and pigs suggests that a porcine model is more suitable for translational research. We aimed at developing a pig model of hypothermia and rewarming that should be minimally invasive, but give maximum information of cardiovascular function. Paramount in this effort would be the use of an indwelling conductance catheter in the left ventricle to extract pressure-volume and contractility data.

Considering animal welfare, we aimed at sedating the pigs deeply, bringing the model close to therapeutic hypothermia as used in human medicine. By avoiding the use of neuromuscular blockers, and by cooling the animals to severe hypothermia, we intended to shed light on aspects of accidental hypothermia as well.

The present study reports that hypothermia below 34°C reduced cardiac contractile functional variables (PRSW and dP/dt_max_) significantly, and that the depressed cardiac function prevailed during rewarming. We demonstrated that post-hypothermic contractile dysfunction is due to an isolated perturbation of systolic function, whereas diastolic function is restored.

## Material and methods

### Animals

Twelve castrated male juvenile pigs (weight 24 to 36 kg) from a native Norwegian stock (norsk landsvin) were used, of whom eight animals were cooled and rewarmed and four animals were kept normothermic as time-matched controls. The animals received humane care in accordance with The Norwegian Animal Welfare Act. The study was approved by The National Animal Research Authority.

The animals were placed in pens for two to five days after arrival to the laboratory animal unit. They were fed twice daily and had free access to water at all times.

### Experimental protocol

**Figure 1:**
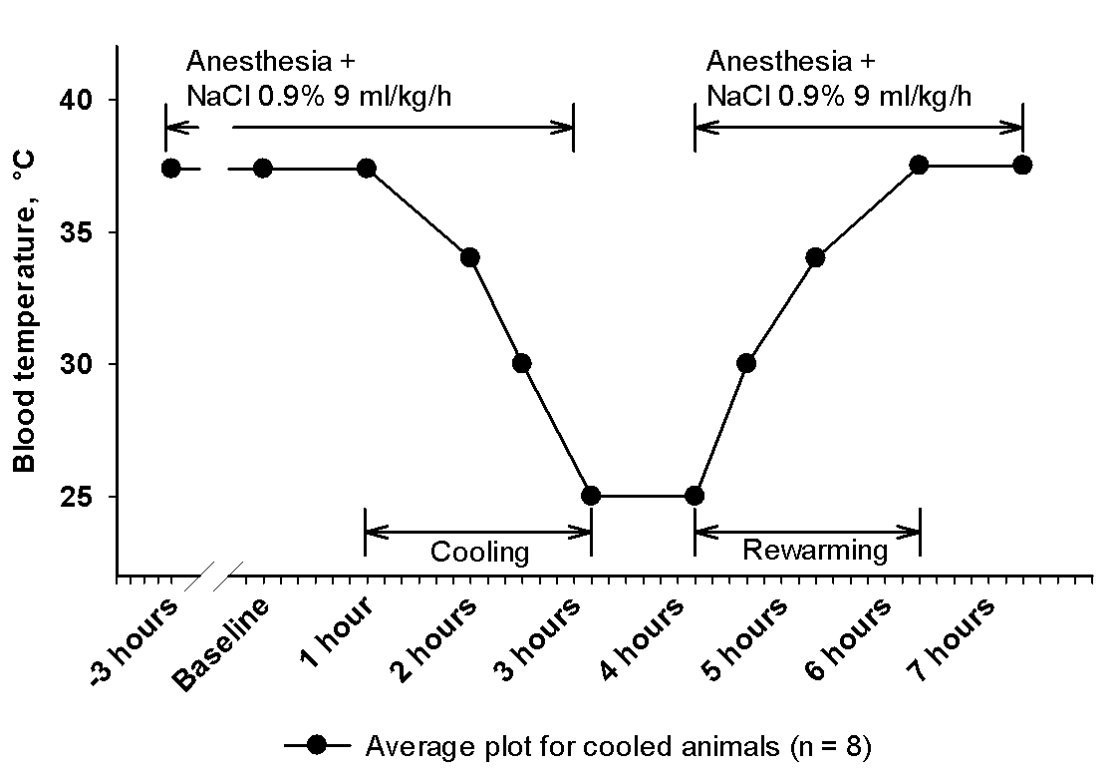
**Protocol layout**.

The overall protocol layout is visualized in Figure 1. Following instrumentation and a one-hour rest, baseline recordings were done, and dopamine infusions at 4, 8 and 16 μg/kg/minute in steps of 15 minutes duration were subsequently started in all animals. The reason for this was that the eight animals subjected to cooling were to serve as controls in another study testing the effects of dopamine during normothermia, severe hypothermia and rewarming. After the dopamine infusion was stopped, the eight animals in the study group were immersion cooled to a core temperature of 25°C. After one hour at this temperature they were rewarmed in warm water to a core temperature of 38°C.

### Anesthesia and instrumentation

After an overnight fast, anesthesia was induced in the pen by an intramuscular bolus of ketamine hydrochloride 20 mg/kg, midazolam 25 mg and atropine 1.0 mg. After transfer to the animal research operating theatre, a catheter was inserted into an ear vein and a bolus injection of fentanyl 10 μg/kg and pentobarbital-sodium 10 mg/kg was given. After tracheostomy a continuous right external jugular vein infusion of fentanyl 20 μg/kg/h, pentobarbital-sodium 4 mg/kg/h and midazolam 0.3 mg/kg/h along with Ringer's acetate 9 ml/kg/h was started and maintained throughout the experiment, except for the one-hour period at 25°C core temperature. After termination of experiments the animals were killed with 20 mmol potassium chloride given as an intravenous bolus. No neuromuscular blockers were used at any time. Animals were maintained on intermittent positive pressure ventilation and a positive end expiratory pressure (PEEP) of 4 cm H_2_O was applied throughout the experiments (Siemens Servo 900 D, Solna, Sweden). FiO_2 _was adjusted to maintain PaO_2 _> 10 kPa, and alveolar ventilation adjusted to keep PaCO_2 _of 4.5 to 6 kPa uncorrected for temperature (α-stat management). Arterial pressure monitoring and blood sampling were obtained via a femoral artery catheter. A 5F thermodilution catheter (Edwards Lifesciences, Irvine, CA, USA) was positioned in the pulmonary artery via the right external jugular vein for pressure monitoring, continuous core temperature recording, cardiac output measurements and for blood sampling. A single dose of 3,000 IU heparin was given after placement of the thermodilution catheter. Via the left carotid artery a 7F dual field, pressure-volume conductance catheter (CD Leycom, Zoetermeer, The Netherlands) was positioned in the left cardiac ventricle for continuous pressure and volume monitoring. To obtain intermittent preload reductions a 7F balloon catheter was positioned in the inferior caval vein via the left femoral vein. A 14F urinary bladder catheter was introduced via a lower abdominal incision for continuous monitoring of urinary output.

### Data sampling

Each data sampling procedure lasted about five minutes and was carried out in the following order: 1) acquisition of electrocardiography (ECG), mean arterial blood pressure (MAP) and central venous pressure (CVP); 2) recording of cardiac output (CO), blood temperature, diuresis and respirator settings; 3) blood sampling from femoral and pulmonary arteries; 4) recording of steady state LV pressure-volume (PV) data, and; 5) recording of PV data during inferior vena cava occlusions. Data sets were collected at baseline, during cooling at core temperatures 34, 30 and 25°C, during maintained hypothermia (25°C) and during rewarming at 30, 34 and 38°C.

### Conductance catheter methods

The conductance catheter placement was guided by LV pressure signals being displayed on a monitor and by advancing the catheter to obtain the maximum number of segments displaying ventricular volumes without causing ventricular arrhythmias. Segments lying outside the ventricle were excluded before each recording. Steady state recordings of PV-loops were performed with the respirator attached. LV contractility at each temperature was determined as the mean value derived from three successive caval occlusions and PV-loop recordings, each time disconnecting the respirator for about 10 sec, and successive respirator attachment and recovery of MAP between occlusions. Data were continuously computed and stored on a Leycom Sigma 5 DF computer (CardioDynamics, San Diego, CA, USA) and later analysed by Circlab software (GTX Medical Software, Zoetermeer, The Netherlands). In analysis, conductance derived CO was corrected against CO measured by thermodilution measurements of cardiac output by a thermodilution computer (Vigilance, Edwards Lifesciences, Irvine, CA, USA) at each temperature step. In this way, there was no need to correct for temperature dependent blood resistivity (rho), as the thermodilution method is independent from conductance catheter recordings and unaffected by rho. During sampling rho was arbitrarily set at the same fixed value on the Leycom Sigma 5 DF computer at all temperatures.

The conductance derived LV end diastolic and systolic volumes (EDV and ESV) resulted from intraventricular conductance and the conductance of surrounding structures, called parallel conductance. Parallel conductance determination by use of repetitive hypertonic (30%) saline infusions at each temperature was not performed, as this would have lead to considerable NaCl accumulation throughout the experiment. Consequently, EDV and ESV in this study do not represent real LV volumes, and no measure of LV ejection fraction could be calculated. However, recording of relative LV volume changes during cooling and rewarming could be performed.

Steady state readings of conductance data gave, in addition to LV volumes, maximum and minimum values of the first derivate of ventricular pressure over time (dP/dt_max_, dP/dt_min_), the time constant of isovolumetric relaxation (Tau) based on a monoexponential decay model and LV end diastolic and systolic pressures (EDP and ESP). Preload recruitable stroke work (PRSW), end systolic elastance (E_es_) and end diastolic pressure volume relationship (EDPRV) were calculated based on PV recordings during abrupt inferior vena cava occlusions. V_0 _was defined by the intercept of the E_es _slope of the x-axis (volume axis).

Arterial elastance (E_a_) was calculated as E_a _= LVESP/stroke volume (SV). Arterial-ventricular coupling ratio was determined by E_a_/E_es_.

At core temperatures below 30°C inferior caval occlusions did not induce changes in PV loops that could be applied to calculate PRSW, EDPVR or E_es_, probably because the heart obtained most of its filling from the superior caval vein at this low-flow state.

### Recording and calculation of hemodynamic variables

ECG from standard leads, heart rate (HR), CVP, MAP, and PAP were continuously displayed on a data monitor and intermittently recorded by a computer program designed at our department using the software package LabVIEW _TM _v.6.1 (National Instruments, Austin, TX, USA). At pre-determined temperatures CO was measured in triplets, by injecting 5 ml precooled saline in the thermodilution catheter positioned in the pulmonary artery. SV and systemic vascular resistance (SVR) were calculated as: SV = CO/HR; SVR = (MAP - CVP) · 80/CO. To index SVR body surface area (BSA) was calculated according to the formula: BSA in m^2 ^= (734 ·Weight ^0.656^): 10,000 [26].

Global delivery and consumption of oxygen (DO_2 _and VO_2_) were calculated as oxygen content in arterial blood · CO, and the difference of arterial and mixed venous oxygen content · CO, respectively.

### Immersion cooling and rewarming

After instrumentation all surgical wounds were sutured in two layers and the animals were laid in a right lateral recumbent position on the operating table. By use of a centrifugal pump (Bio-Medicus, Eden Prairie, MN, USA), and a heat-exchanger (Stöckert Normo/hypothermie, Munich, Germany), cold water (5°C) circulated the hollow operating table and a tarpaulin tub surrounding the animal. The upper left side of the animal was covered with ice slush and irrigated by cold water leaving two-thirds of the dependent animal submerged. The head was placed on a cushion and not immersed or covered with ice slush. At 26°C core temperature cold water circulation was discontinued, the tub drained for water and ice slush, and core temperature subsequently dropped to approximately 25°C in all animals. To prevent core temperature from a further drop, small amounts of warm water was added to the tub. Rewarming was achieved by circulating the operating table and the tub, and by irrigating the upper left side of the animal, with hot water (40 to 42°C, measured in the afferent water hose) till rewarming (38°C) was accomplished.

### Biochemical analyses

#### Catecholamines

Blood with heparin (4 IU/ml), reduced glutathione (4.5 mM) and EGTA (5 mM) was kept on ice/water for maximally 30 minutes before plasma was obtained by centrifugation (1.000 × g) for 20 minutes at 4°C. Samples were stored at -80°C awaiting analysis.

Plasma samples (1 to 2 ml) were spiked with known concentrations of the internal standard (DHBA = dihydroxy-benzylamine) and added 1 ml 2 M Tris-EDTA buffer (pH 8.7). The catecholamines were adsorbed onto alumina (10 mg). After aspiration of plasma/buffer, the alumina was washed three times with bi-distilled water (1 ml). The catecholamines were eluted from the alumina with a mixture (100 μl) comprising acetic acid (175 mM), sodium bisulfide (9 mM) and EDTA (0.7 mM). After whirling and centrifugation, the aquous phase was aspirated and transferred to the autoinjector (Dionex ASV-100, Dionex, Sunnyvale, CA, USA).

Dopamine, norepinephrine and epinephrine were separated by HPLC (Dionex P680, Dionex ASV-100, Dionex, Sunnyvale, CA, USA; Chromsystems analytical column and eluent, Chromsystems Instruments&Chemicals GmbH, Munich, Germany) and their concentrations determined with an electrochemical detector (ESA Coulochem, III, ESA, Chelmsford, MA, USA). The analyses were performed at ambient temperature with a flow of 1.2 ml/ml.

#### Other analyses

Hemoglobin (Hb) measurements, and arterial and mixed venous blood gases were analysed on a blood gas analyser (Rapid lab, Chiron Diagnostics, Emeryville, CA, USA) uncorrected for temperature. Blood samples for serum analysis were put on ice, quickly centrifuged and the serum was then quickly frozen and kept at -80°C awaiting analysis. Tumor necrosis factor alpha (TNF-α) was analysed by the quantitative sandwich enzyme immunoassay technique (Quantikine^®^, R&D Systems, Inc., Minneapolis, MN, USA). Troponin T (TnT), ASAT, ALAT and albumin were analysed by the sandwich method of electrochemiluminescence, UV-test with pyridoxal phosphate activation, and a colorimetric end point method (Modular, Roche Diagnostics, Rotkreuz, Switzerland).

### Statistical analyses

Statistical analyses were performed using SigmaPlot statistical software version 9 - 11 (Systat Software Inc. (SSI), Richmond, CA, USA). Intragroup comparisons were performed by one-way repeated measures analysis of variance, or by paired sample Student's *t *test when only single comparisons were made. For comparisons between groups, the Student's *t *test was used if data showed normal distribution, otherwise the Mann-Whitney rank sum test was used. Level of significance was set at *P *≤0.05. Data are presented as mean ± SEM. Statistically significant changes are referred to as simply significant changes for the sake of convenience.

## Results

### Cooling and rewarming observations

Immersion cooling to 25°C lasted 125 ± 15 minutes giving a cooling rate of 6.8 ± 0.7°C/h. Rewarming lasted 133 ± 6 minutes, giving a rewarming rate of 6.0 ± 0.3°C/h. The phenomenon referred to as 'afterdrop', a decrease in core temperature after onset of surface warming, was not observed in any pigs. Visible shivering took place in all pigs at the start of cooling, but subsided with progressive cooling and was absent at 25°C. Little, if any, shivering was observed during and after rewarming.

### Effects of cooling, steady state severe hypothermia, and rewarming on cardiovascular function

#### Mild hypothermia

**Figure 2:**
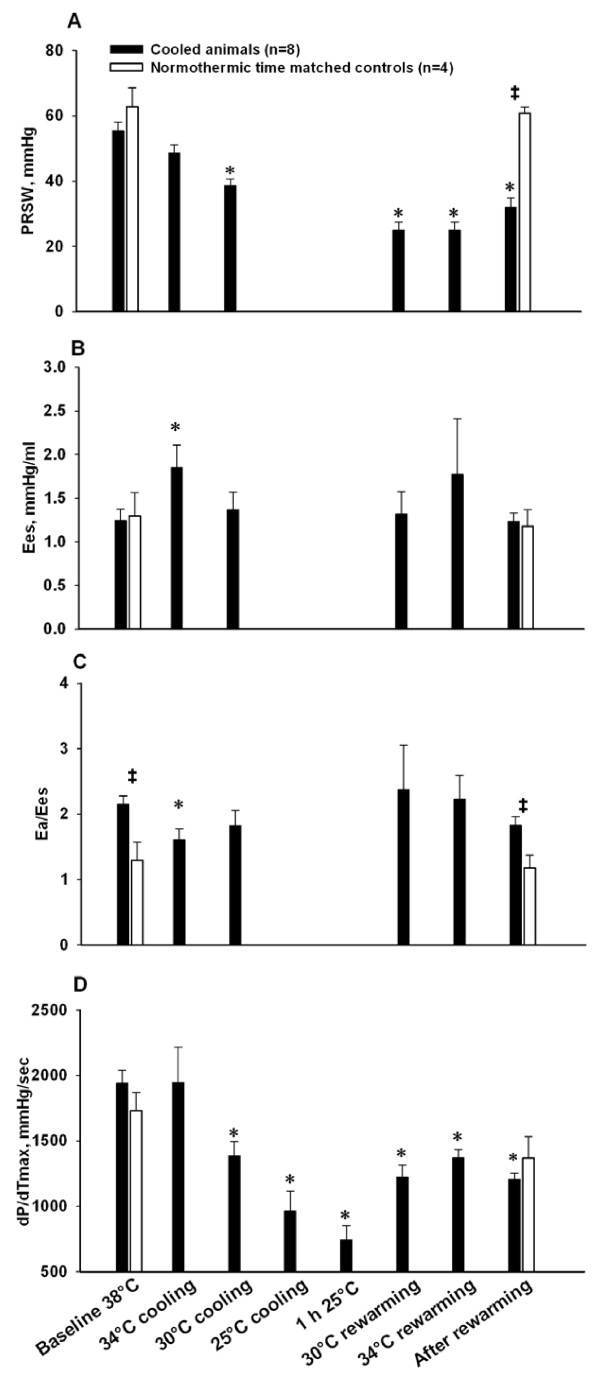
**Left ventricular contractility data**. **A**. Preload recruitable stroke work (PRSW); **B**. end systolic elastance (E_es_); **C**. arterial-ventricular coupling ratio (E_a_/E_es_); **D**. maximal acceleration of pressure in the cardiac cycle (dP/dt_max_). *Significantly different from baseline (*P *≤0.05). **‡ **Significant difference between cooled animals and time matched normothermic controls (*P *≤0.05).

**Figure 3:**
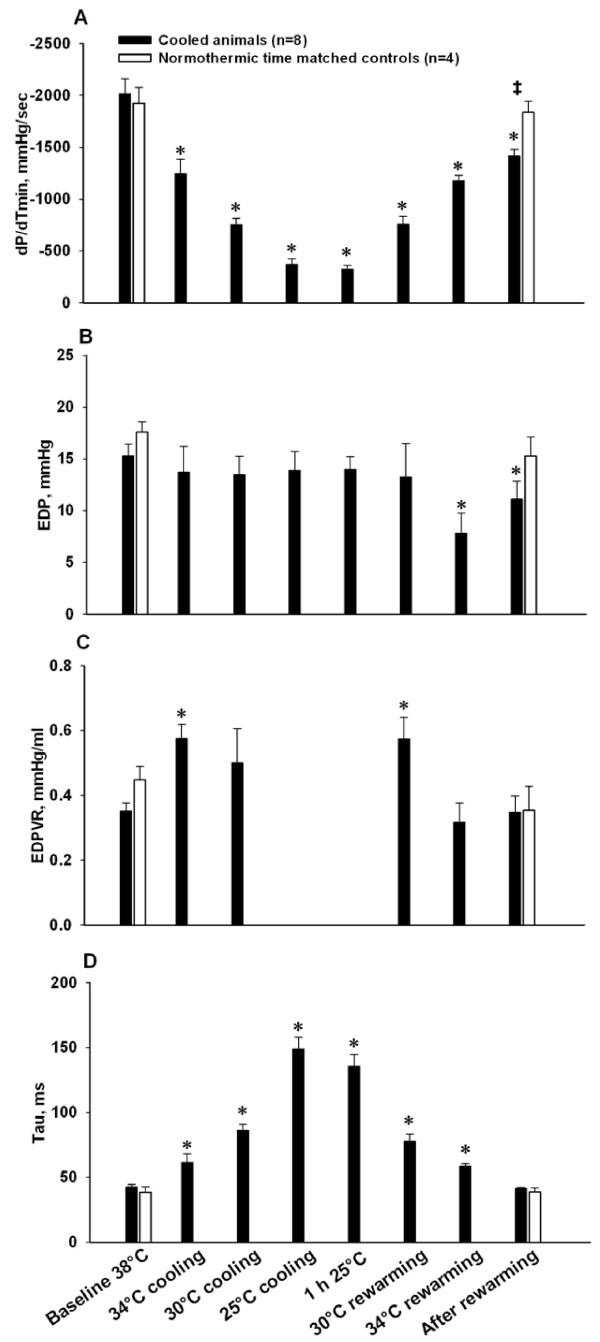
**Indexes of left ventricular diastolic function**. **A**. Maximal deceleration of pressure in the cardiac cycle (dP/dt_min_); **B**. end diastolic pressure (EDP); **C**. end diastolic pressure volume relationship (EDPVR); **D**. isovolumetric relaxation time (Tau). *Significantly different from baseline (*P *≤0.05). **‡ **Significant difference between cooled animals and time matched normothermic controls (*P *≤0.05).

**Figure 4:**
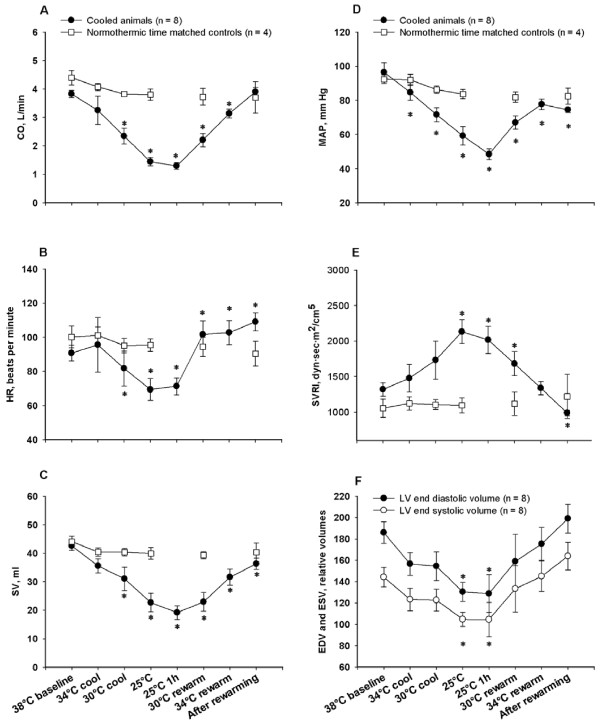
**General hemodynamic recordings**. **A**. Cardiac output (CO); **B**. heart rate (HR); **C**. stroke volume (SV); **D**. mean arterial pressure (MAP); **E**. systemic vascular resistance index (SVRI); **F**. end diastolic and systolic volumes (EDV and ESV). *Significantly different from baseline (*P *≤0.05).

E_es _(Figure 2B) as the sole indicator of LV contractility, increased significantly at 34°C, as did indexes of diastolic function, Tau (Figure 3D) and EDPVR (Figure 3C). dP/dt_min _(Figure 3A) decreased significantly (maximal deceleration decreased). MAP (Figure 4D) and E_a_/E_es _(Figure 3C) decreased significantly at 34°C. All other hemodynamic variables were statistically unaffected by cooling to 34°C.

#### Cooling below 34°C

Indexes of LV contractility PRSW (Figure 2A) and dP/dt_max _(Figure 2D) decreased significantly during cooling below 34°C, while E_es _and E_a_/E_es _(Figure 2C) were statistically unaffected. V_0 _(not shown) was statistically indifferent to moderate and severe cooling, as were EDP (Figure 3B) and EDPVR. EDV, ESV (Figure 4F) and CVP (not shown) decreased during cooling, reaching statistical significance at 25°C. dP/dt_min _was statistically reduced in a linear pattern during cooling, whereas Tau increased in a pattern reciprocal to the dP/dt_min _curve.

CO (Figure 4A), HR (Figure 4B), SV (Figure 4C), and MAP all decreased significantly in a linear way below 34°C. SVRI (Figure 4E) increased in a nearly similar pattern, reaching significance at 25°C. Except for cold-induced bradycardia, no arrhythmias were encountered until severe hypothermia was established.

#### One hour steady state hypothermia at 25°C

No PV data derived from inferior caval occlusions were available at this temperature. Values for dP/dt_max_, dP/dt_min_, CO, SV and HR reached their nadir during the 1 h period at 25°C, while Tau reached its highest value, as did SVRI.

During stable severe hypothermia sinus bradycardia and various idioventricular arrhythmias were seen. The ECG did not show atrial fibrillation nor the so called J-wave or Osborn wave, both characteristics of severe hypothermia in humans [27] in any pig. In four out of eight pigs the phenomenon of mechanical (or pulsus) alternans [28] occurred; that is, a reduced stroke volume observed on the conductance monitor every other heart beat in the absence of corresponding abnormalities in the ECG. One animal got ventricular fibrillation (VF) that was successfully defibrillated to an organized rhythm during the one-hour period at 25°C.

#### Rewarming and post-hypothermic results

During rewarming PRSW and dP/dt_max _were significantly lowered when compared to corresponding temperatures during cooling. HR made an abrupt and significant increase during rewarming from 25 to 30°C, and remained significantly elevated at a stable level above this temperature. The other variables that were reduced by cooling tended to approach corresponding values during rewarming in a nearly mirror image pattern.

After rewarming PRSW, dP/dt_max, _dP/dt_min _, SV, MAP, EDP and SVRI remained significantly decreased compared to pre-hypothermic baseline values. CVP, EDV and ESV returned to pre-hypothermic controls. While post-hypothermic E_es _was statistically unchanged, V_0 _was significantly increased after rewarming.

Due to a significant increase in HR, post-hypothermic CO returned to within pre-hypothermic values. Tau and EDPVR, having been increased during rewarming, returned to control. In all animals cardiac rhythm spontaneously returned to sinus rhythm and mechanical alternans disappeared during rewarming.

### Effects of cooling, steady state severe hypothermia and rewarming on other variables

**Figure 5:**
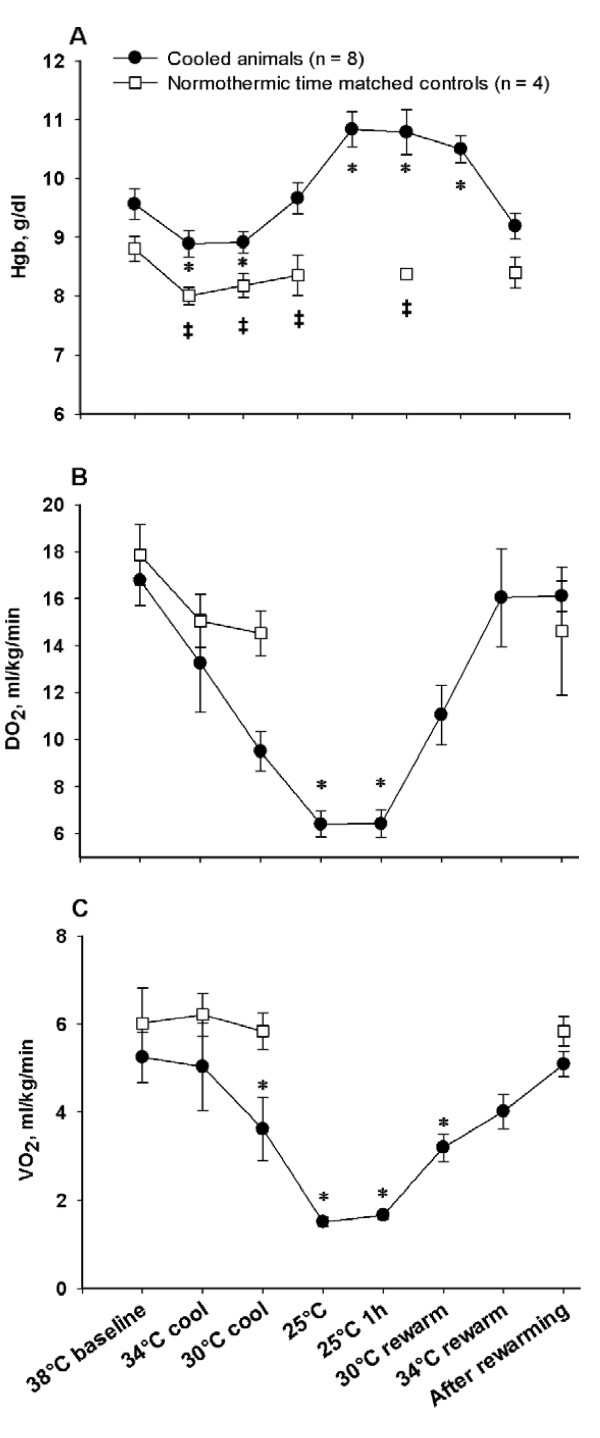
**Oxygen variables**. **A**. Blood hemoglobin concentration (Hb); **B**. global delivery of oxygen (DO_2_); **C**. global consumption of oxygen (VO_2_). *Significantly different from baseline (*P *≤0.05). **‡ **Significant difference between cooled animals and time matched normothermic controls (*P *≤0.05) in Figure 5A.

**Figure 6:**
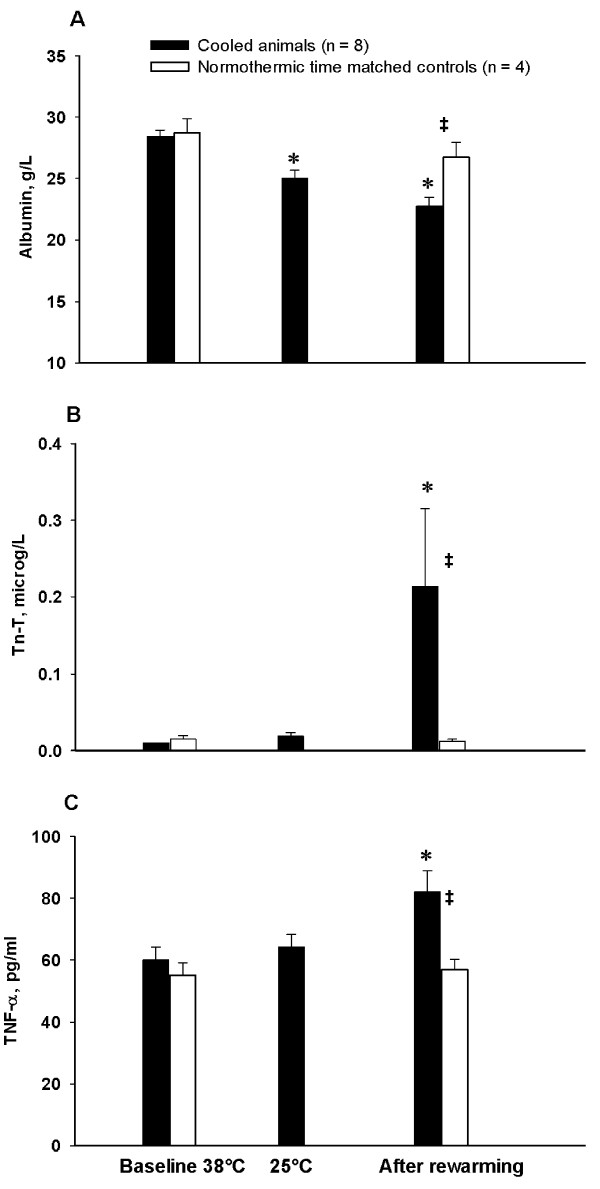
**Biochemical variables**. **A**. Serum albumin concentration; **B**. serum troponin T concentration (TnT); **C**. serum tumor necrosis factor alpha concentration (TNF-α). *Significantly different from baseline (*P *≤0.05). **‡ **Significant difference between cooled animals and time matched normothermic controls (*P *≤0.05).

Hemoglobin (Hb) concentration (Figure 5A) showed a biphasic pattern, decreasing significantly during cooling and increasing significantly during severe hypothermia and rewarming. Oxygen content of arterial and mixed venous blood (not shown) followed a pattern nearly synchronous with the Hb curve, increasing statistically during severe hypothermia. The global delivery and consumption of oxygen (DO_2 _and VO_2_, Figure 5B, C) were reduced by 61 ± 4% and 68 ± 6% respectively during cooling, giving corresponding reductions of 4.7 ± 0.3% and 5.2 ± 0.4% per degree C. While DO_2 _correlated with temperature in a linear manner, the reduction in VO_2 _was just about 1% per degree C in the temperature interval between 38 and 34°C and about 7.8% per degree C from 34 to 25°C, reflecting that the relationship between VO_2 _and decrease in temperature took the form of a negative exponential function. During rewarming DO_2 _returned to pre-hypothermic baseline values following a mirror image of the pattern during cooling, while VO_2 _normalized in a more linear way than during cooling. Albumin values (Figure 6A) decreased significantly during cooling and remained significantly reduced after rewarming. Significant increases in both troponin T (TnT, Figure 6B) and TNF-α (Figure 6C) serum concentrations were seen after rewarming, both in contrast to their own baseline values and to time matched controls. Serum concentrations of dopamine, epinephrine and nor-epinephrine (not shown) were statistically unchanged from pre-hypothermic values throughout the experiments. Diuretic output (not shown) was not affected by temperature throughout experiments.

## Discussion

The present study demonstrates that severe hypothermia (30 to 25°C) reduced cardiac contractile functional variables (PRSW and dP/dt_max_) significantly, and that the depressed cardiac function prevailed during rewarming. After rewarming we found that post-hypothermic contractile dysfunction is due to isolated perturbation of systolic function, whereas diastolic function is restored.

Our anesthetized animals, showing no endogenous catecholamine response during cooling or rewarming, clearly differ from awake animals subjected to surface cooling [8], bringing the actual model close to the clinical hypothermia setting. As no neuromuscular blockers were administered, initial shivering during cooling, well known from accidental and clinical hypothermia [4,29], was present. Shivering occurred in spite of the apparently deep anesthetic state. This may be a species related phenomenon, as shivering in therapeutic hypothermia in man most often can be controlled with various sedative drugs [7].

### LV systolic function

Data collected at 34°C suggest a possibly increased inotropy at this temperature, as E_es _was increased and PRSW and dP/dt_max _were unaffected. Progressive cooling from 34°C to severe hypothermia induced a reduction in LV contractile indexes PRSW and dP/dt_max_, that, together with reduced SV, suggests reduced systolic function. Moreover, the significantly reduced PRSW and dP/dt_max _during and after rewarming compared with corresponding temperatures before and during cooling, imply a reduction in LV contractility that progresses with the duration of hypothermia, and prevails after rewarming. This is in accordance with findings of increasing hypothermia-induced cardiac failure reported in rats when duration of severe hypothermia was increased from one to five hours [30] and with experimental findings that post-hypothermic mortality due to circulatory failure in dogs and rodents increased with the duration of exposure to severe hypothermia [22,31].

While the complete pathophysiology of hypothermia-induced cardiac failure is not known, it seems that among other factors cytosolic Ca^2+ ^overload is involved, possibly via a temperature-dependent dysfunction of ion transport [32]. Hypothermia-induced cardiac failure shares similarities with myocardial stunning in that both conditions may involve intracellular Ca^2+ ^overload that may partly be attributed to hypothermia-induced inhibition of the Na^+^/K^+^-ATPase in the sarcolemma, partly to impaired clearance of free cytosolic Ca^2+^. While the proposed mechanism in hypothermia is a temperature-dependent dysfunction of ion transport mechanisms, the concept of myocardial stunning is invariably linked to the ischemia-reperfusion syndrome where dysfunction of ion transport mechanisms due to lack of ATP, together with production of reactive oxygen species, causes Ca^2+ ^overload and modification of contractile proteins [33].

The reason why E_es_, as opposed to the other LV contractility indexes, was statistically unchanged at moderate and severe hypothermia as well as during and after rewarming is not clear. One factor making E_es _potentially unreliable during hypothermia may be that as core temperature is reduced, the effect of caval vein occlusions diminishes, thus making ESPVR slope determination unreliable. Hypothermia-induced bradycardia contributes to fewer ESPVR points during occlusions, another factor that may affect the accuracy of the slope. Furthermore, E_es _has been proven to be an unreliable contractility index in itself in experimental heart failure, as significant increases in E_es _were encountered in a pig model of stunning, acute ischemic and endotoxemic heart failure despite obvious other indications (significant decreases in CO, dP/dt_max _and MAP) of depressed myocardial function [34]. The increases in E_es _were accompanied with significant increases in V_0_, which apparently resulted in steep E_es _slopes [34]. This parallels to the finding of a post-hypothermic significant increase in V_0 _in the present study. In previous experiments in dogs at our laboratory aimed at determining LV cardiac contractility during cooling and rewarming it was concluded that PRSW appeared to be a more robust index of contractility than E_es _during hypothermia [18].

One could argue that, in the absence of VCO-derived contractility data at 25°C, single beat estimates of contractility could have replaced traditional deloading variables. However, *in vivo *single beat contractility estimations in pig have been shown to be unreliable and no better than dP/dt_max _in predicting LV contractility [35].

### LV diastolic function

During hypothermia changes in diastolic functional variables were measured. Most prominent was the increase in indexes of isovolumetric relaxation, Tau and dP/dt_min_, indicating a temperature-dependent slowing of sarcoplasmic reticulum (SR) function that together with elevated intracellular Ca^2+ ^concentration delayed clearance of cytosolic Ca^2+^. This finding corresponds well with the fact that diastolic Ca^2+ ^extrusion depends mainly on SR Ca^2+ ^pump activity, also during hypothermia [36], and that enzyme kinetics of the Ca^2+ ^pump is highly temperature dependent (Q_10 _effect). The passive, late diastolic phase, the filling phase, was less compromised during hypothermia as indicated by a modest increase in EDPVR, or stiffness, in the present experiment. The effects of change in temperature on the SR Ca^2+ ^pump are demonstrated by the return to control of Tau after rewarming.

The post-hypothermic reduction of dP/dt_min _(reduction in maximal deceleration) may imply an early diastolic dysfunction which remained from the hypothermic period. However, Tau and EDPVR returned to pre-hypothermic levels, EDV was statistically unchanged and EDP was even reduced after rewarming. We interpret the post-hypothermic increase in dP/dt_min _as resulting from the concomitantly decreased MAP, and suggest that post-hypothermic early, as well as late, diastolic function is normalized after rewarming. This is in accordance with previous findings that diastolic function was restored in post-hypothermic core cooled dogs [18] and rats [24].

If the post-hypothermic state is characterized by systolic failure with maintained diastolic function, this clearly makes post-hypothermic cardiac dysfunction different from myocardial stunning, where not only systolic, but also diastolic dysfunction is an invariable finding [37].

### Heart rate

Increasing HR from 80 to 120/minute by pacing in normothermic humans before hypothermic coronary bypass grafting increased LV contractility [38]. When the same procedure was repeated during cardio-pulmonary bypass at 33°C, pacing led to decreased contractility [38]. Also the increase in LV contractility seen when cooling of rabbit hearts *in vitro *was lost when normothermic HR was maintained by pacing during hypothermia [13]. Thus, an artificial increase in HR during hypothermia may be detrimental to LV contractility. In the present study, we measured an abrupt increase in HR from 25 to 30°C during rewarming. At this time point, PRSW was significantly lower than at 30°C during cooling. This raises the question; did the abrupt increase in HR cause a concomitant reduction of PRSW, or was the increase in HR a spontaneous compensatory reflex aimed at elevating cardiac contractility? As discussed above, our data support the understanding that the duration of severe hypothermia *per se *leads to decreased LV contractility, and we interpret the increase in HR as a compensatory mechanism whereby the organism normalized CO and DO_2 _in the presence of a decreased SV. According to Opie (1998), 'LV failure exists (...) when myocardial ejection is impaired because the inotropic state is depressed' [39]. Hypothermia-induced cardiac dysfunction may be perceived as a variant of acute LV failure. Clinically, increased heart rate is a common sign in acute heart failure [40], as a consequence of increased sympathetic activity to compensate for LV failure [39]. Experimentally, statistically significant elevation of plasma catecholamines is associated with pathologic reduction of myocardial contractility [41]. In the present study, however, there was no detectable increase in plasma catecholamine levels. It may still be that receptors in the heart, aortic arch and carotid sinus triggered by decreased pressure and/or flow activated brain stem neurons, leading to efferent nervous stimulation of the sinoatrial node, materialized in the present experiment by a significant increase in HR.

### Vascular tone

The post-hypothermic reductions of MAP and SVR open for at least two possible interpretations; either i) we see a variant of the somewhat imprecise term 'rewarming shock', characterized by reduced cardiac function and drop in blood pressure [42], or ii) the reduced SVR is an adaptation to the reduced myocardial contractility.

In recent years, an integrated view of the interaction between vascular tone and cardiac contractility has been pursued through the study of the coupling between arterial and cardiac elastance, expressed as the E_a_/E_es _coupling ratio [43]. E_a _in itself, being the ratio between LVESP/SV, is not a variable solely derived from the vascular system, and as such does encompass more than SVR. Effective coupling of heart to artery may be defined as the optimal transfer of blood from heart to periphery without excessive changes in blood pressure, and it has been reported that an E_a_/E_es _coupling ratio of 0.6 to 1.2 represents a near optimal relation between work and efficiency [44]. When systolic heart failure (low E_es_) is accompanied by high arterial elastance (high E_a_), the E_a_/E_es _is elevated and the unfavorable situation of afterload mismatch occurs [44]. In the present study post-hypothermic E_a_/E_es _was statistically unchanged from baseline. If MAP and SVR had remained unchanged from pre-hypothermic values, E_a _would have been increased, possibly leading to an E_a_/E_es _ratio significantly increased in an unfavorable direction. In our opinion the reduced post-hypothermic SVR, in the presence of reduced LV contractility and SV, does not necessarily express 'rewarming shock', but may imply an optimized arterial-ventricular coupling ratio that together with increased HR facilitates more physiologic CO and DO_2._

### Cardiac arrhythmias

The low incidence of ventricular fibrillation (VF) in our model may be ascribed as species-specific. However, it is likely that the deep anesthetic state protected against cardiac arrhythmias, as catecholamines, especially epinephrine, from both endogenous and exogenous sources have been proven to illicit VF during experimental surface cooling [45,46].

### Oxygen variables

There is no evidence in our material that global oxygen consumption at any time was dependent on oxygen delivery. Two facts support this statement: i) oxygen content of mixed venous blood increased during hypothermia, and ii) the difference in oxygen content between arterial and mixed venous blood in the hypothermic animals was no different from time-matched normothermic controls. Previous experiments in our laboratory have demonstrated that increased oxygen extraction from mammalian arterial blood is possible at temperatures as low as 15°C [30]. Further, while the present study relates to global variables, it has been shown that in dogs cooled to 25°C, no myocardial oxygen or substrate deficits was present [47].

In the present experiments animals shivered during initial cooling and this fact may explain why oxygen consumption was only marginally reduced during this phase of the protocol. In the absence of shivering, the relationship between core temperature and oxygen consumption would probably have been linear rather than taking the observed form of a negative exponential function.

### Fluctuations in blood volume

The temperature-dependent fluctuations in Hb concentration throughout the experiment could not be attributed to periodic blood loss, as bleeding from instrumentation was minimal and blood sampling was evenly distributed. Also, Hb concentration changes were unrelated to diuresis, as diuretic output was constant throughout experiments. More likely, changes in Hb concentration were caused by fluctuations in plasma volume by other mechanisms, as has been reported in the time course of surface cooling [48,49] and rewarming [50]. Our findings of temporarily decreased CVP, EDV and ESV during hypothermia support the assumption that plasma volume was reduced in hypothermia and returned to normal during rewarming.

### Biochemical markers

Animals subjected to cooling and rewarming showed a small, but statistically significant increase in TnT that was not present in time-matched normothermic controls. One may suggest that the observed increase in TnT was due to Ca^2+ ^mediated activation of intracellular proteases that degrade troponins in the same manner as has been suggested to take place during myocardial stunning [33].

Post-hypothermic increase in TNF-α and reduced plasma albumin concentration in our experiment may indicate activation of inflammatory mediators by cooling-rewarming, as has been shown in both surface- and endovascular - cooling in the same species [48,51].

### Cooling and rewarming rates

A cooling rate of about 6°C per hour, as in the present experiments, is faster than in induced therapeutic hypothermia [7], but appears comparable to that in victims of accidental immersion hypothermia. The rewarming rate of 6.0 ± 0.3°C/h in our experiment is, however, much faster than that achieved by active external rewarming in clinical settings, where immersion in hot water is seldom used. Kornberger *et al*. refer to 15 victims of severe accidental hypothermia rewarmed by forced air at a mean rate of 1.7°C per hour [20]. Furthermore, they reported no incidents of core temperature afterdrop after the start of external rewarming [20], which is in accordance with findings in this study. Our results do not support that fear of core temperature afterdrop should dictate a particular slow rewarming rate.

## Conclusions

Surface cooling to 25°C followed by rewarming resulted in reduced systolic, but not diastolic, LV function. The post-hypothermic increase in heart rate and the reduced systemic vascular resistance are interpreted as adaptive measures by the organism to compensate for a hypothermia-induced mild left ventricular cardiac failure.

## Key messages

• Cardiac left ventricular contractility decreases during surface cooling below 34°C.

• Cardiac systolic function is depressed after rewarming from 25°C.

• Cardiac diastolic function is restored after rewarming from 25°C.

• Post-hypothermic cardiac output is maintained by increased heart rate.

• Decreased post-hypothermic systemic vascular resistance may be adaptive measure.

## Abbreviations

CO: cardiac output; CVP: central venous pressure; DO_2_: global delivery of oxygen; dP/dt_max_: maximal acceleration of pressure in the cardiac cycle; dP/dt_min_: maximal deceleration of pressure in the cardiac cycle; E_a_: arterial elastance; E_a_/E_es_: arterial-ventricular coupling ratio; E_es_: end systolic elastance: slope of the linear ESPVR for a family of PV-loops during VCO; EDP: end diastolic pressure; EDPVR: end diastolic pressure volume relationship; EDV: end diastolic volume: in the present paper consisting of LV end diastolic volume and the volume of surrounding structures; ESP: end systolic pressure; ESV: end systolic volume: in the present paper consisting of LV end systolic volume and the volume of surrounding structures; ESPVR: end systolic pressure volume relationship; Hb: hemoglobin; HR: heart rate; LV: left ventricular; MAP: mean arterial pressure; PRSW: preload recruitable stroke work; PV: pressure-volume; SV: stroke volume; SVR(I): systemic vascular resistance (index); Tau: isovolumetric relaxation time; TNF-α: serum tumour necrosis factor alpha; TnT: serum cardiac troponin T; V_0_: volume axis intercept of the ESPVR slope determined by PV-loops during VCO; VCO: vena cava occlusion: abrupt occlusion of the inferior caval vein to obtain PV-loops; VF: ventricular fibrillation; VO_2_: global consumption of oxygen.

## Competing interests

The authors declare that they have no competing interests.

## Authors' contributions

OMF conducted the experiments and, together with TT, designed the protocol, discussed the data and wrote the manuscript. OJH took part in protocol and scientific discussions, assisted in carrying out experiments and contributed to the writing process. TK took part in carrying out experiments and processing and interpretation of data. TMG took part in protocol discussions and in carrying out experiments. All authors read and approved the final manuscript.
